# Co-Benefits of China’s Carbon Emissions Trading Scheme: Impact Mechanism and Spillover Effect

**DOI:** 10.3390/ijerph20053792

**Published:** 2023-02-21

**Authors:** Zijian Liu, Lian Cai, Yabin Zhang

**Affiliations:** School of Economics & Trade, Hunan University, Changsha 410079, China

**Keywords:** carbon emissions trading scheme, co-benefits, DID model, spillover effect

## Abstract

Based on the panel data of 281 prefecture-level cities in China, from 2007 to 2017, we empirically explore the co-benefits of the carbon emissions trading scheme. We found that the carbon emissions trading scheme effectively achieved the coordinated control of carbon dioxide and air pollutants, by improving the green production level of the pilot areas, reducing the regional industrial output, and promoting the upgrading of the industrial structure. In terms of heterogeneity, the emissions trading scheme shows obvious urban location and level heterogeneity, in terms of coordinated control. The synergistic emission reduction effects of eastern and central cities are significantly better than those of cities in central and western regions and non-central cities. It has also had positive spillover effects on the surrounding cities of the pilot areas, but pollution levels in farther areas may have increased due to possible “pollution shelter problems”.

## 1. Introduction

China’s economy has achieved rapid development in the past 40 years and is now one of the largest economies in the world. However, there are more and more serious environmental problems, especially the emission of CO_2_ and air pollutants. The nation’s air quality standards were exceeded by 53.4% of Chinese cities. Serious air problems will cause harm to people’s physical and mental health, and also incur huge governance costs [1]. Therefore, the Chinese government is faced with the dual task of controlling CO_2_ and air pollutant emissions, which means that in order to achieve the goal of minimizing policy costs, co-benefits must be considered.

The co-benefit comes from the concept of ancillary benefits, proposed by Ayres and Walter in 1991 [2], which is used to indicate that the reduction in greenhouse gases can reduce the emission of other air pollutants, and was expanded to co-benefits by the IPCC in 2001 [3], which is used to measure the reduction in other pollutants beyond the main target of an environmental policy. Nowadays, carbon trading has become a common international means to control greenhouse gas emissions. Countries around the world are also trying to improve their carbon trading systems to combat climate change [4,5]. The effect of this measure on controlling the emissions of other air pollutants should also be considered.

Based on this concept, the USEPA launched the ICAP project in 2000, and took the lead in researching synergistic effects of policies on air pollution control. Since then, the environmental policies’ co-benefits have gradually become an important basis for evaluating an environmental policy’s effects [6,7,8]. The reasons why China’s environmental policy must consider co-benefits come from two aspects. One is the similar emission characteristics of greenhouse gases and other major atmospheric pollutants (sulfur oxides, nitrogen oxides, soot, etc.), which have the same root, homologous and simultaneous characteristics in their emission process, making their governance and control have a very high synergy. Recently, more and more researchers have called for coordinated efforts to improve air quality and combat climate change [9,10,11]. On the other hand, China’s coal-dominated energy structure also makes for greater co-benefits, because coal contains more sulfur and nitrogen than oil, thus producing more air pollutants during combustion [6,7].

To encourage enterprises to reduce CO_2_ emissions, the Chinese government officially launched China’s emissions trading scheme (ETS) in 2011, and in October of that year, pilots were launched in Hubei, Guangdong, Beijing, Shanghai, Tianjin, and Chongqing. Formal trading began in June 2013. In Beijing Municipality’s carbon emission trading management (trial), the specified goal of the policy was to reduce CO_2_ emissions, while also reducing emissions of other air pollutants [10].

The existing literature focuses on policies specifically targeting the control of air pollutants [12,13,14,15,16,17], for example, Meng et al. examined the effect of household fuel policies in northern China [17]. But in recent years, the Chinese government has implemented multiple environmental policies targeting CO_2_ emissions; it is necessary to examine whether these yield co-benefits by reducing emissions of air pollutants. In fact, many studies have investigated the co-benefits of environmental policies in Europe and the United States [15,16,17,18], while research on the Chinese ETS still focuses on its carbon reduction effect and the promotion of low-carbon technological innovation [19,20,21,22,23,24,25], or only studies the impact on a single air pollutant indicator. For example, Liu et al. used the DID model to test the impact of China’s ETS on PM_2.5_ [8], they found that China’s ETS significantly reduced PM_2.5_ concentrations in the pilot areas, and the effect was strongest in the summer. Chang et al. used a CGE model to analyze the relationship between air quality and health, and predicted that by around 2030, the provincial PM_2.5_ concentration will drop by 4–20% [26]. Cheng et al. found that China’s ETS could reduce SO_2_ emissions in Guangdong Province by 12.4% by 2020 [27].

Most previous research has only paid attention to the cooperative control of China’s ETS on a specific air pollutant, but has rarely measured the cooperative control effect of China’s ETS on air pollutants as a whole. This may deprive us of the opportunity to look at the collaborative control of CO_2_ and air pollutants as a whole. Therefore, to prevent the underestimation of co-benefits, it is necessary to construct a comprehensive index of air pollutants to measure the co-benefits of China’s ETS [28,29,30,31,32]. Also, the existing literature evaluating co-control effectiveness is mostly based on both national and provincial levels, the small number of studies that have focused on cities, only focus on whether the synergistic control effect for PM_2.5_ of a city has been achieved [33]. As the most important units for carbon and local air pollutants emission accounting, statistics and management, it is essential to study multiple cities, as this will lead to more convincing conclusions about the co-benefits of China’s ETS.

In addition, most of the literatures only focuses on whether China’s ETS has realized the cooperative control of CO_2_ and air pollutants, without an in-depth analysis of the influencing mechanism behind it. This makes it very difficult to understand how China’s ETS works. Meanwhile, the spillover effects of China’s ETS on the neighborhood, which is an important part of the policy’s effect, are rarely reported in the existing literature [33], this has also prevented us from fully evaluating the effect of China’s ETS.

In order to make up for the shortcomings of the existing research, our contribution is threefold: Firstly, using city-level data, which was rarely used in the existing literature, we evaluated the synergistic control effect of China’s ETS on different types of air pollutants (AP) at the overall level; secondly, we provide an in-depth analysis of how ETS works, which helps us to better understand how ETS achieves the co-benefits of reducing emissions of CO_2_ and other air pollutants; finally, we assess the spillover effects of China’s ETS on the surrounding region and present new findings about the effects of China’s ETS policies.

## 2. Research Design

### 2.1. The DID Model

Our research is based on the following difference-in-difference model as follows:

lnAP_i,t_, lnCO_2_ = β_0_ + β_1_treated_i_ ∗ time_t_ + γX_i,t_ + α_i_ + u_t_ + ε_i,t_ (1)

where treated_i_ and time_t_ are dummy variables. Since 2013, China has carried out ETS pilots in seven provinces and cities, including Beijing, Shanghai, Tianjin, Chongqing, Hubei, Guangdong, and Shenzhen. Although they included industries in different orders, this does not affect our estimates, as we only focus on co-benefits that differ between the pilot and non-pilot regions. So we set treated_i_ = 1 when the city belonged to an ETS pilot area and when the date was after 2014 (time_t_ = 1). City fixed effects, α_i_, are also included, controlling for all unobserved factors that differ only across cities, and time fixed effects, u_t_, controlling for all unobserved factors that differ only across time. ε_i,t_ is the error term.

### 2.2. Variable Selection

First, based on the research of Zhang et al. [31], we constructed a new index to comprehensively measure the emission of air pollutants. The formula is as follows:

AP_i,t_ = Σef ∗ QAP_i,t,f_ = αSO_2i,t_ + βQNOx_i,t_ + γPM_10i,t_ + δCO_i,t_ + εVOCs_i,t_

In the formula, AP_i,t_ represents the equivalent value of air pollutants emitted by city i at time t, and ef represents the coefficient of air pollutant f in converting the equivalent value of air pollutants. The specific values of ef are shown in Table 1. The coefficient for different kinds of air pollutants, such as α for SO_2_, can be determined with reference to the emission concentration standards of air pollutants, environment tax rates, and the costs of pollutant discharge damage [32]. Although there is no universal conversion standard for various pollutants, theoretically, the coefficient should reflect the pollutant control priority in the decision-making process, based on marginal social welfare loss. A commonly accepted rule is that the coefficients must reveal their externalities, and should be assigned by the impacts of pollutants on living organisms and human health. Thus, the coefficient ef here is decided based on official environmental tax rates, carbon prices, and global warming potential [31,32].

The selection of carbon trading pilot cities was not completely random, and there must be many differences between pilot cities and non-pilot cities. Therefore, in order to avoid endogeneity problems, variables that may have determined whether a city was selected as a pilot city for ETS were included in the model. Following analysis of the existing literature, and relevant official documents, the control variables for the final constituency in this paper included: (1) GDP per capita (PGDP); (2) industrial structure (IS), i.e., the ratio of the secondary industry’s GDP to the total GDP; (3) the development level of the service industry (SL), i.e., the tertiary industry’s value as a proportion of the total GDP; (4) industrial structure advanced (IU), i.e., the ratio of the output value of the tertiary industry to that of the secondary industry; (5) comprehensive utilization rate of industrial solid waste (CUR); and (6) the city’s industrial electricity consumption (IPC). We argue that most cities with a high degree of industrialization have various types of industrial enterprises, and cities with a higher level of economic development have entered the stage of seeking high-quality development earlier than other cities, and these cities are more likely to be selected as ETS pilot areas. At the same time, cities with a developed service industry have many advantages. For example, cities with a developed education industry have better human capital, and a developed financial industry can better solve the financing problems that may arise when enterprises achieve carbon reduction. Such cities are more capable of coping with changes brought about by ETS and are more likely to have been selected as pilot cities. In addition, if a locality has a high comprehensive utilization rate of industrial solid waste, it means that the region has certain experience in pollution prevention and control, and it was also more likely to be selected as a pilot city for this policy.

We summarize the selected variables and how they were measured in Table 2.

### 2.3. Data Resources

The data of CO_2_ and air pollutant emissions come from the *China Energy Statistical Yearbook* and the relevant research of the IPCC, and the control variable data come from the *China Urban Statistical Yearbook*. After deleting missing data and abnormal data, we finally obtained data from 281 prefecture-level cities in China from 2007 to 2017, with which to test the ETS’s co-benefits.

We deleted the city of Suizhou from the final regression sample. Among the data we collected, large parts of the discharge data of various pollutants in Suizhou were missing, and no detailed data were obtained before 2012. As one of the core explained variables in this paper, too short a period of sample years would cause an estimation bias of the DID model. The degree of participation of Suizhou in the carbon trading market is not high, so we believe that, on the whole, eliminating the sample of Suizhou did not have a substantial impact on the overall results.

### 2.4. Descriptive Analysis

Through descriptive analysis, we can have a general understanding of the changes in CO_2_ and AP before and after the implementation of the carbon trading pilot policy. Table 3 shows the results. The difference between pilot and non-pilot cities before the implementation of the policy in 2014 was very small. After 2014, CO_2_ emissions in the pilot cities dropped significantly, and AP also decreased a lot, indicating that the reduction in carbon dioxide emissions also contributed to the reduction in air pollutants through the synergistic control effect. In contrast, AP of non-pilot cities did not change much.

## 3. Results

### 3.1. Base Results: The Effect on Co-Benefits

Base estimates of ETS’s co-benefits are shown in Table 4. Columns (1–3) first test the carbon emission reduction. The results show that, when other variables are controlled, the ETS has significantly reduced CO_2_ emissions in pilot cities, by about 4.8%. In columns (4–6), we performed regression analysis according to Equation (1), and controlled the linear and nonlinear time trends of these variables in turn. The results show that China’s pilot carbon trading policy still significantly promotes the reduction in AP, and the decrease rate is about 6.91%, with other variables kept unchanged. This result is similar to the research conclusions of other scholars, who used samples from other countries [21,31]; China’s ETS has achieved co-benefits by reducing the emission of CO_2_ and other air pollutants at the same time. However, because of China’s industrial-oriented industrial structure, this effect seems to be smaller than that of the USA’s carbon policies, which have been estimated to have offset at least 26% of policy costs [7].

### 3.2. Parallel Trend Test

In the base results, we identified the synergistic control effect of the ETS, but an important assumption of the difference-in-difference model is that there is no significant difference in the development trend of the control group and the treatment group before the policy implementation. Based on this, we constructed the following model on the basis of Formula (1) for the parallel trend test:

(2) $$lnAP_{i,t},lnCO2_{i,t}=\beta_{0}+\sum_{t=-3}^{2}\beta_{t}\left(1\left\{Yr=t\right\}\times treated_{i}\right)+\gamma X_{i,t}+\alpha_{i}+u_{t}+\varepsilon_{i,t}$$

**Figure 1:**
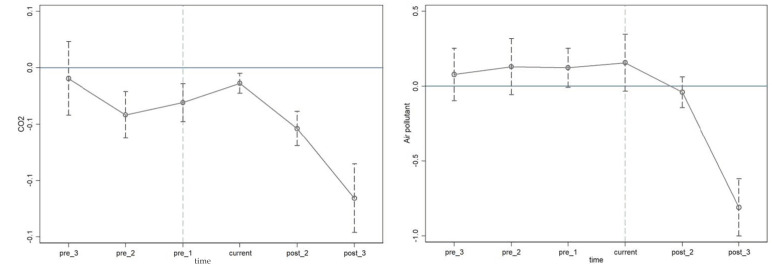
Parallel trend test for CO_2_ and AP.

Figure 1 is made with the regression coefficient by year as the vertical axis, and time as the horizontal axis. The results show that before 2014, the treatment group’s, and the control group’s, CO_2_ and AP emissions had the same development trends, so the results in this paper passed the parallel trend test.

### 3.3. Robustness Test

#### 3.3.1. Placebo Test

**Figure 2:**
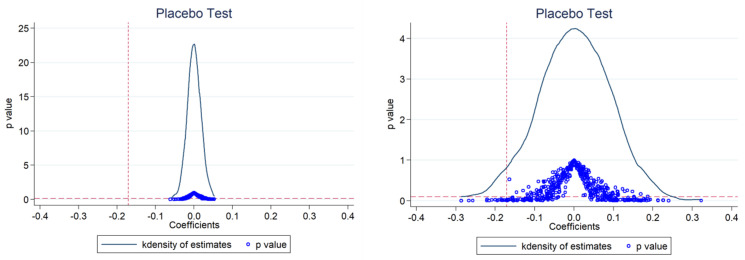
Placebo test for CO_2_ and AP.

Referring to the research of La Ferrara et al. and Li et al. [34,35], we constructed a city-level random experiment by randomly choosing ETS pilot cities, and performing a regression according to Equation (1). After repeating the above process 1000 times, we plotted the distribution of the estimated coefficients of the interaction term treated_i_ ∗ time_t_ (Figure 2). The results show that the coefficients of interaction term estimated by the false regression, arr, is mostly distributed around 0 and not significant, indicating that the model in this paper does not miss key variables, and the regression results are robust.

#### 3.3.2. Propensity Score Matching (PSM)

In the selection of pilot cities for carbon trading, some cities may have stronger collaborative control capabilities. Therefore, it was not appropriate to select cities with large differences in environmental governance capabilities for the control group. In order to minimize the initial difference between the treatment and control groups, we further adopted propensity score matching (PSM), to test the causal relationship between the ETS and co-benefits when cities with similar conditions were used as the control group.

Referring to the selected control variables in Equation (1), we established a Logit model of whether a city became a carbon trading pilot object, and obtained the probability of each city receiving treatment (being selected as a carbon trading pilot city), that is, the propensity score, and adopted the method of radius matching, set the matching radius to 0.05, and performed basic regression on the matched samples. Columns (1) and (2) of Table 5 report the results after adding the nonlinear time trend effect of the control variables. The results show that treated_i_ ∗ time_t_’s coefficient is not significantly different from that of the basic results above. After controlling for other variables, the implementation of the carbon trading pilot policy still reduced CO_2_ emissions by about 4.74%, and reduced AP by about 7.41%. The regression results in columns (1) and (2) show that when the explained variable is AP, the coefficient is larger than that of the baseline regression, which indicates that after using a more accurate matching sample, the effect of the carbon trading pilot policy on coordinated control is stronger.

#### 3.3.3. Alternative Calculation Method

Due to the imperfect construction and slow development of China’s emission rights trading market, and it only being limited to a few regions and enterprises, there are also problems such as unreasonable initial emission rights allocations and unreasonable price mechanisms, and the secondary market lacks vitality. There are also great differences in the trading of emission rights, which makes it impossible to select an appropriate transaction price of emission rights to determine the equivalent value coefficient, so it is impossible to construct an appropriate pollution equivalent index. In the previous analysis, it was mentioned that the coal-dominated energy structure means that China has a strong synergistic control effect when dealing with air pollution and climate change. Therefore, under this background, we selected the most important harmful air pollutant produced by coal combustion, SO_2_, replaced the air pollutant emission equivalent in the synergistic control effect index with the emission of SO_2_ in the sample city, and ran the regression again. Column (3) of Table 5 shows the regression results. The results are basically consistent with the above, and the stability of the model is verified again.

#### 3.3.4. Winsorization

In the base regression, there may be cities with very good or very bad carbon and air pollutants reduction performance, and it is possible that the existence of these samples could affect the final conclusion. In order to exclude the influence of extreme values, we carried out a 1% two-sided tailed treatment on the explained variables, and the specific results are shown in Table 5. The data in columns (4) and (5) show that the regression results after the tail reduction treatment are similar to the basic regression, which further confirms our conclusion.

#### 3.3.5. Policy Interference

Many other environmental policies were implemented during our sample year. National environmental policies (e.g., the 12th Five-Year Plan and the Action Plan for air pollution prevention and control) should not have affected our estimation results, because they affected all sample cities, including the treatment group and the control group. Such policies will not have biased the estimation results of DID. However, those environmental policies only targeted at some specific cities, such as the Blue Sky protection policy, and the promotion policy of new energy logistics vehicles in Inner Mongolia, may have caused biases in our estimation results. Therefore, we excluded those cities that independently implemented targeted environmental policies from the sample (e.g., Blue Sky Defense). The regression results are shown in column (6) and (7) of Table 5. The data show that our core conclusion is still valid, and removing these samples has no obvious impact on the results.

### 3.4. Heterogeneity Analysis

#### 3.4.1. City Location

China’s regional economic development is unbalanced. The eastern region has the advantage of coastal areas and convenient transportation, which can more easily absorb foreign advanced technology. In contrast, the infrastructure, technological foundation, and human capital of the central and western regions lag behind the eastern regions as a whole. Does this urban location difference have an impact on the implementation of the ETS? In order to explore this question, we classified the treatment groups according to the division of the eastern, central, and western provinces and cities by the National Bureau of Statistics, and performed a grouped regression according to Equation (1). Columns (1–4) of Table 6 show the regression results. The study found that urban location affects the synergistic control effect of carbon trading policies. Co-benefits in eastern cities are bigger than in central and western cities. This may be because the central and western regions have the geographical advantages of coastal areas and convenient transportation, which can more easily absorb foreign advanced technologies. In contrast, the infrastructure, scientific, and technological foundation and human capital of the central and western regions lag behind the eastern regions on the whole.

#### 3.4.2. City Level

In recent years, in order to achieve more effective regional development, China has gradually implemented the strategy of urban agglomeration. The central city can effectively connect with other cities at all levels, depending on transportation and information infrastructure. Therefore, central cities have a stronger ability to obtain various production factors, and there are also differences in factor endowments from non-central cities. Central cities have a stronger ability to regulate production, better human capital, and more efficient research and development of cleaner production technologies. In this paper, we also ran a grouped regression, and the results are shown in columns (5–8) of Table 6. The results show that the synergistic control effect in central cities is significantly different from other cities. The possible reason is that the central cities have better innovation factors and resource conditions, thus, it is easier to promote the development of cleaner production technology.

## 4. Impact Mechanism

From the previous empirical analysis, it can be seen that the ETS has a significant synergistic control effect, indicating that the pilot of this policy can control carbon dioxide emissions while also controlling other air pollutants. To further investigate how the carbon trading pilot policy achieves the synergistic control effect, we discuss its mechanism from three perspectives.

First of all, for the government, environmental governance and economic construction are an important part of government performance appraisal. Therefore, in order to improve performance, local governments will increase subsidies for technological innovation of industrial enterprises and help enterprises develop cleaner production technologies; under the influence of the ETS, the optimal choice of enterprises is also green innovation. The enterprises’ green innovation investments and government subsidies will promote the improvement of the green production level in pilot areas [33], which will yield co-benefits.

Industrial structure optimization is also an important channel for ETS to take effect [31,32,33,34], and for industrial enterprises, the carbon trading pilot policy mainly affects their production costs. So for high-carbon-emission enterprises, on the one hand, expanding the production scale requires the additional purchase of carbon dioxide emission rights beyond the quota in the carbon trading market, which undoubtedly increases the production cost of the enterprise; on the other hand, low-carbon-emission companies have more funds to conduct research and development of cleaner production technologies, and the production cost gap between them and high-carbon-emission companies will further widen. The above two factors will cause some enterprises with backward production technology to choose to withdraw from the market due to cost pressure, realize the optimization of the industrial structure, and then realize the coordinated control of carbon dioxide and air pollutants in the region.

Existing companies may also reduce production plans, to balance the rising cost of carbon trading pilot policies [36,37,38,39]. That is to say, the reduction in carbon dioxide and air pollutants in pilot cities is not necessarily due to the reduction in carbon intensity, but because the policy has resulted in a reduction in local economic output [40]. Based on the above analysis, we selected the level of green production, the degree of industrial structure optimization, and the output of the industrial sector as the mechanism variables to test the synergistic control effect of ETS, and constructed the following model to test the relationship between the mechanism variables and carbon trading pilot policies:

Y_i,t_ = β_0_ + β_1_treated_i_ ∗ time_t_ + γX_i,t_ + α_i_ + u_t_ + ε_i,t_ (3)

In the above analysis, Y_i,t_ represents the mechanism variable in which the level of green production is measured, by green total factor productivity (GTFP); the optimization of industrial structure is investigated from two aspects, labor productivity and inter-industry ratio, and calculated according to the formula AIS_i,t_ = v_i,t_ ∗ LP_i,t_, where v_i,t_ represents the proportion of secondary industry output value in the GDP of city i at time t, and LP_i,t_ represents the labor productivity of city i at time t; local industrial production is measured using the same-named indicator in the *China Urban Statistical Yearbook*, the term treated_i_ ∗ time_t_ has the same meaning as the interaction term in Formula (1), X_i,t_ represents a series of control variables, and the model also adds the city fixed effect and year fixed effect, and ε_i,t_ is the error term. Table 7 shows the regression results. Column (1) examines the impact of the carbon trading pilot policies on local green production levels, column (2) examines their impact on local industrial output value, and column (3) examines their impact on industrial structure optimization. The distribution of key coefficients in the regression results is basically consistent with the theoretical analysis above: the ETS has finally yield co-benefits by improving the level of green production, promoting the optimization of the industrial structure, and reducing the economic output of local industries.

## 5. Additional Impacts: Spatial Spillovers

Environmental regulation has a significant impact on industrial pollution in surrounding cities, so the spatial spillover effects of policies must be considered. As far as the ETS is concerned, on the one hand, enterprises in the pilot cities may migrate to the surrounding non-pilot cities to continue production due to cost reasons, which will increase the emissions of carbon dioxide and air pollutants in the surrounding cities, and become “pollution shelters”, showing a negative spillover effect; on the other hand, market-based environmental regulation has a significant positive spillover effect on neighboring areas, such as significantly reducing the PM_2.5_ concentration in the surrounding area [8]. Therefore, we built a model to explore the spillover effects of ETS on neighboring areas.

Clarke et al. proposed a method to identify spillover effects in a two-difference model [41]. First, the adjacent areas were grouped according to the distance from the pilot area. Considering that the spatial spillover effect will gradually attenuate with the increase in distance, we took 350 km as the farthest distance with the spillover effect, and divided the adjacent areas into five groups. r1 represents a city with a distance of 0–150 km from the pilot area; r2 represents a city with a distance of 150–200 km from the pilot area; r3 represents a city with a distance of 200–250 km from the pilot area; r4 represents a city with a distance of 250–300 km from the pilot area; r5 represents a city with a distance of 300–350 km from the pilot area, and the following model was set:

$$Y_{i,t}=\mu +\tau D_{i,1}+\overset{5}{\underset{k=1}{\sum }}\gamma_{k}{R^{k}}_{i,1}+\delta t+\alpha D_{i,t}+\overset{5}{\underset{k=1}{\sum }}\beta_{k}{R^{k}}_{i,1}+Control_{i,t}$$

Y_i,t_ represents the explained variable. We selected the CO_2_ emissions and the air pollutants emissions, AP, of the adjacent area, and took the logarithm; D_i,1_ represents the dummy variable, representing whether it is a pilot city for ETS.; Rk_i_,_1_ means a dummy variable, representing whether the distance from the pilot area is within the group of r_k_ (k = 1, 2, 3, 4, 5); t represents the time variable; D_i,t_ is the treated_i_ ∗ time_t_ item in Formula (1), when the sample is a pilot city and the time is after 2014, it is assigned a value of 1, and the rest are 0; R^k^_i,t_ is assigned a value of 1 when the sample is a neighboring area with a distance from the pilot area within the r_k_ group, and the time is after 2014, and 0 in other cases; Control_i,t_ represents the control variables and, according to the method of Liu et al. [8], we select the same control variables in the base regression. The coefficient β_k_ is the influence of the spillover effect. Table 8 shows the regression results. We gradually added different distance groups in columns (2) to (6) to show the change in β_k_. The analysis shows that the carbon trading pilot has had a significant positive spillover effect on the co-benefits of adjacent areas within a distance of 150 km. This may be because the pilot areas include economically developed central cities. Driven by regional integration and industrial agglomeration, the level of green production in adjacent areas has been improved and the industrial structure has been upgraded. At the same time, China’s ETS has also increased the emission of air pollutants from cities within 50 km of the pilot area. This indicates that market-based environmental regulations have significant positive spillover effects on neighboring areas, which may be because the pilot area has promoted green production reforms of the surrounding area. However, because enterprises in the pilot cities may move to the surrounding non-pilot cities to continue production, due to cost reasons, the air pollutant emissions in the surrounding cities have increased, becoming “pollution shelters”, and the air pollutant emission levels in distant areas have increased.

## 6. Conclusions

We take the ETS implementation city as the main research object, to explore whether there are co-benefits. The results of this paper show that: (1) Overall, the ETS has a significant synergistic control effect, and this conclusion is shown to be valid in a series of tests, such as a parallel trend test and placebo test; (2) in terms of heterogeneity, the ETS shows obvious urban location heterogeneity in terms of coordinated control, the synergistic emission reduction effects of eastern and central cities are better than those of central and western cities and non-central cities; (3) in terms of the impact mechanism, the carbon trading pilot policy improves green production level, promotes the optimization of industrial structure, reduces local industrial economic output, and finally yields co-benefits; (4) China’s ETS has a significant positive spillover effect on the surrounding cities of the pilot areas, but pollution levels in more distant areas are likely to increase due to possible “pollution shelter problems”.

Based on the conclusions of our research, we suggest that: at the current stage, making full use of the synergistic control effect is a necessary condition for realizing China’s environmental governance goals and ecological vision. The synergistic control effect of environmental policies on different types of air pollutants can make our policies gain more, which enables us to effectively save the cost of policy implementation, and thus make environmental governance more efficient. At the same time, governments must recognize that the same policy implemented in different regions may have different effects. Environmental policies also have an impact on surrounding areas, which should be taken into account. A comprehensive assessment of environmental policies is an essential step towards improving the environment.

Our study is based on city-level data from China, which is more accurate than the provincial-level data used in previous studies. However, the ETS is mainly aimed at companies that emit various types of air pollutants. Future research could use enterprise-level data to further explore the effect of the policy, and distinguish its impact on different industries. In particular, China’s ETS pilot program has been gradually rolled out by industry in pilot cities, and the effect of the policy on different industries should be studied. The efficiency and stability of the market, especially the volatility of the carbon price, should be examined. In addition, our discussion of spillover effects is relatively simple, and future studies could use more models, such as a spatial error model, spatial lag model, and spatial panel regression, to make a more comprehensive study of the spillover effects of this policy. Finally, future research could examine the effects of season and wind direction on the policy’s effectiveness (as Liu et al. [8] did in their research).

## Figures and Tables

**Table 1:** Values of coefficients of air pollutants.

Types	Coefficient	Value	Source	Explanation
SO_2_	α	1/0.95	According to the “Environmental Protection Tax Law” in China: “Taxable Pollutants and Equivalent Value Table”, each pollution equivalent value is “0.95 kg SO_2_, 0.95 kg NOx, 16.7 kg CO, 2.18 kg soot……”, according to which the conversion coefficients of various air pollutants to AP_i,t_ can be obtained, and the PM_10_ is valued according to the equivalent conversion coefficient of “smoke and dust”.	According to “The schedule of Tax items and tax rates in the Environmental Protection Tax Law of China”, per unit of local air pollutants equivalence is equal to 0.95 kg SO_2_, 0.95 kg NOx, 2.18 kg PM_10_, 16.7 kg CO, 0.95 kg VOCs.
NOx	β	1/0.95		
PM_10_	γ	1/2.18		
CO	δ	1/16.7		
VOCs	ε	1/0.95		

**Table 2:** Measures and variables.

Dependent Variables	
lnCO_2_	Log of CO_2_ emissions at the city level
lnAP	Log of the comprehensive index of air pollutant emission at the city level according to Table 1
Independent Variables	
treated_i_ ∗ time_t_	treated_i_ = 1, when the city belongs to an ETS pilot area and when the time is after 2014 (time_t_ = 1)
Controls	
PGDP	GDP per capita
IS	The ratio of the secondary industry’s GDP to the total GDP
SL	The tertiary industry’s total value as a proportion of the total GDP
IU	The ratio of the output value of the tertiary industry to that of the secondary industry
CUR	Comprehensive utilization rate of industrial solid waste
IPC	The city’s industrial electricity consumption

**Table 3:** Statistical analysis.

Variables	Means before 2014	Means after 2014	SE	Min	Max	Observations
CO_2_	27.7675	27.2106	42.7714	6.5380	230.7117	36
CO_2_ *	27.8164	27.5764	20.1961	2.0907	129.6009	245
AP	11.5898	11.2308	0.85545	8.8662	13.5844	36
AP *	11.4745	11.4481	0.9169	4.6806	14.7594	245

Note: * represents the data of non-pilot cities.

**Table 4:** Regression results.

Variables	ln(CO_2_)			ln(AP)		
	(1)	(2)	(3)	(4)	(5)	(6)
treated_i_ ∗ time_t_	−0.0656 ***	−0.0668 ***	−0.0480 ***	−0.0719 **	−0.0695 *	−0.0691 **
	(0.0095)	(0.0168)	(0.0091)	(0.0267)	(0.0308)	(0.0258)
Con_	3.3044 ***	2.7598 ***	3.1887 ***	11.4376 ***	10.1839	11.4322
	(0.0931)	(0.0063)	(0.3559)	(0.3166)	(0.4123)	(0.3258)
Fixed effect	No	No	Yes	No	No	Yes
Controls × t	No	Yes	Yes	No	Yes	Yes
Controls × t^2^	No	Yes	Yes	No	Yes	Yes
Controls × t^3^	No	Yes	Yes	No	Yes	Yes
N	2565	2565	2565	2205	2205	2205
R-squared	0.9908	0.9744	0.9927	0.6775	0.6835	0.7197

Note: Reported in parentheses are the robust standard errors that are clustered at the city level. *** *p* < 0.01. ** *p* < 0.05. * *p* < 0.1.

**Table 5:** Robustness test.

Variables	PSM-DID		Variable Substitution	Winsorization		Policy Interference	
	lnCO_2_ (1)	lnAP (2)	lnSO_2_ (3)	lnCO_2_ (4)	lnAP (5)	lnCO_2_ (6)	lnAP (7)
treated_i_ ∗ time_t_	−0.0474 ***	−0.0741 **	−0.0595 **	−0.0586 ***	−0.0539 *	−0.0506 **	−0.0767 *
	(0.0124)	(0.0301)	(0.0192)	(0.0168)	(0.0283)	(0.0207)	(0.0355)
Con_	2.208 ***	10.30 ***	10.79 ***	2.761 ***	11.48 ***	3.0286 ***	10.07 ***
	(0.385)	(0.469)	(0.386)	(0.0062)	(0.222)	(0.0097)	(0.461)
Fixed effect	Yes	Yes	Yes	Yes	Yes	Yes	Yes
Controls × t	Yes	Yes	Yes	Yes	Yes	Yes	Yes
Controls × t^2^	Yes	Yes	Yes	Yes	Yes	Yes	Yes
Controls × t^3^	Yes	Yes	Yes	Yes	Yes	Yes	Yes
Observations	2491	2149	2230	2012	1897	1536	1780
R-squared	0.827	0.727	0.724	0.789	0.739	0.767	0.727

Note: Reported in parentheses are the robust standard errors that are clustered at the city level. *** *p* < 0.01. ** *p* < 0.05. * *p* < 0.1.

**Table 6:** Heterogeneity analysis results.

Variables	Cities in Central and Western Regions		Cities in Eastern Region		Non Central City		Central City	
	lnCO_2_ (1)	lnAP (2)	lnCO_2_ (3)	lnAP (4)	lnCO_2_ (5)	lnAP (6)	lnCO_2_ (7)	lnAP (8)
treated_i_ ∗ time_t_	−0.0391 *	−0.0704 **	−0.0604 ***	−0.0755 **	−0.0445 *	−0.0695 *	−0.0656 **	−0.0752 **
	(0.0203)	(0.0304)	(0.0145)	(0.0299)	(0.0215)	(0.0308)	(0.0259)	(0.0292)
Con_	3.0972 ***	10.18 ***	2.9118 ***	10.08 ***	3.225 ***	9.76 ***	3.302 ***	10.13 ***
	(0.1824)	(0.412)	(0.1706)	(0.459)	(0.159)	(0.262)	(0.159)	(0.3690)
Fixed effect	Yes	Yes	Yes	Yes	Yes	Yes	Yes	Yes
Controls	Yes	Yes	Yes	Yes	Yes	Yes	Yes	Yes
N	2312	2003	2491	2156	2005	2005	2265	2149
R-squared	0.9918	0.726	0.8127	0.726	0.991	0.697	0.992	0.73

Note: After examination, the coefficients of the grouped regressions are comparable and the differences between them are significant. Reported in parentheses are the robust standard errors that are clustered at the city level. *** *p* < 0.01. ** *p* < 0.05. * *p* < 0.1.

**Table 7:** Mechanism test results.

Variables	GTFP	Gross Output	AIS
	(1)	(2)	(3)
treated ∗ time_t_	0.0066 *	−0.1487 *	249.2 **
	(0.0040)	(0.0911)	(126.6)
Con_	0.957 ***	1.183 **	−534.7
	(0.0288)	(0.461)	(397.7)
Fixed effects	Yes	Yes	Yes
Controls	Yes	Yes	Yes
N	2176	2645	2186

Note: Reported in parentheses are the robust standard errors that are clustered at the city level. *** *p* < 0.01. ** *p* < 0.05. * *p* < 0.1.

**Table 8:** Spillover effects.

Variables	lnCO2	lnAP				
	(1)	(2)	(3)	(4)	(5)	(6)
treated_i_ ∗ time_t_	−0.152 *** (0.0158)	−0.0808 (0.0567)	−0.107 (0.0606)	−0.0878 (0.0575)	−0.111 * (0.0491)	−0.115 ** (0.0491)
β1	−0.0833 *** (0.0248)	−0.183 *** (0.0388)	−0.158 *** (0.0423)	−0.177 *** (0.0434)	−0.154 *** (0.0383)	−0.150 *** (0.0383)
β2	−0.0768 *** (0.0223)		0.288 ** (0.0915)	0.268 ** (0.0892)	0.289 ** (0.0871)	0.293 *** (0.0817)
β3	−0.0798 *** (0.0176)			−0.199 (0.119)	−0.179 (0.120)	−0.174 (0.124)
β4	−0.0984*** (0.0142)				0.0967 ** (0.0369)	0.101 ** (0.0398)
β5	−0.0762 *** (0.0196)					0.0232 (0.0453)
Controls	Yes	Yes	Yes	Yes	Yes	Yes
FE	Yes	Yes	Yes	Yes	Yes	Yes
N	2565	2203	2203	2203	2203	2203
R-squared	0.694	0.730	0.731	0.731	0.731	0.731

Note: Reported in parentheses are the robust standard errors that are clustered at the city level. *** *p* < 0.01. ** *p* < 0.05. * *p* < 0.1.

## Data Availability

Data available for request.
